# Co-occurring mental illness, drug use, and medical multimorbidity among lesbian, gay, and bisexual middle-aged and older adults in the United States: a nationally representative study

**DOI:** 10.1186/s12889-020-09210-6

**Published:** 2020-08-04

**Authors:** Benjamin H. Han, Dustin T. Duncan, Mauricio Arcila-Mesa, Joseph J. Palamar

**Affiliations:** 1 Department of Population Health, New York University School of Medicine, 550 First Avenue, New York, NY 10016 USA; 2 Division of Geriatric Medicine and Palliative Care, Department of Medicine, New York University School of Medicine, 550 First Avenue, New York, NY 10016 USA; 3 Department of Epidemiology, Mailman School of Public Health, Columbia University, 722 W 168th St, New York, NY 10032 USA

**Keywords:** Chronic disease, Multimorbidity, Geriatrics, Sexual minorities

## Abstract

**Background:**

Older lesbian, gay, and bisexual (LGB) adults are an underserved and understudied population that experience specific health disparities. The intersection of aging and chronic medical disease with a higher risk for substance use and mental illness may place older LGB adults at risk for co-occurring conditions and resulting comorbidity. Understanding multimorbidity among older LGB adults may help inform interventions to reduce disparities in health outcomes.

**Methods:**

Data come from the 2015 to 2017 National Surveys on Drug Use and Health (*n* = 25,880). We first determined whether sexual orientation was associated with reporting: past-year drug use, mental illness, and/or 2 or more chronic medical diseases. We then determined whether sexual orientation was associated with reporting co-occurrence of these conditions. This was done using multivariable logistic regression. Analyses were stratified by gender.

**Results:**

Compared to heterosexual men, gay men were at increased odds for reporting 2 or more chronic medical diseases (adjusted odds ratio [aOR] = 2.18, 95% confidence interval [CI] = 1.48, 3.21), and gay (aOR = 1.79, 95% CI = 1.09, 2.93) and bisexual men (aOR = 3.53, 95% CI = 2.03, 6.14) were at increased odds for reporting mental illness. Gay men (aOR = 2.95, 95CI = 1.60, 5.49) and bisexual men (aOR = 2.84, 95% CI = 1.58, 5.08) were at increased odds of reporting co-occurring conditions. Compared to heterosexual women, bisexual women were at increased odds for past-year drug use (aOR = 4.20, 95% CI = 2.55, 6.93), reporting mental illness (aOR = 1.94, 95% CI = 1.03, 3.67), and reporting co-occurring conditions (aOR = 3.25, 95% = 1.60, 6.62).

**Conclusions:**

Middle-aged and older LGB adults in the United States are at high risk for experiencing co-occurring drug use, mental illness, and/or medical multimorbidity. Interventions for older sexual minority populations are needed to reduce disparities.

## Background

Existing research demonstrates sexual minority disparities in mental illness, drug use (including substance use disorders [SUDs]), chronic health conditions, and health-related behaviors among adults [1–6]. Awareness of the health burdens experienced by sexual minorities, including lesbian, gay, bisexual, and transgender (LGBT) adults has increased, with their inclusion in the US Department of Health and Human Services’ *Healthy People 2020* as an goal to improve the health of sexual minorities [7].

However, despite the aging of the US population, middle-aged and older LGBT adults remain an understudied group with notable gaps in aging research for this population [8]. An estimated 2 % of the US population age ≥ 65 identify as LGBT, and this population is likely to grow with a population increase of older adults and as more older adults may feel comfortable answering questions about sexual minority status and gender identity [9]. The health of middle-aged and older LGBT adults should be a priority as aging is the main risk factor for the presence of multiple chronic conditions, declines in physical and cognitive function, and increases in healthcare utilization [10]. One study examining data from the 2013–2014 National Health Interview Survey (NHIS) found that among adults age ≥ 50 who identify as lesbian, gay, or bisexual (LGB), older lesbian or bisexual women were more likely to have more chronic diseases while older gay or bisexual men were more likely to have angina pectoris or cancer, and disability was higher among LGB older adults for both identities compared to older heterosexuals [3]. Other studies have shown higher rates of anxiety, depression, illicit opioid use, prescription tranquilizer misuse, and SUDs among middle-aged and older LGBT adults [9, 11, 12].

Conversely, another study from the 2013–2014 NHIS of adults age 65 and older found sexual minorities were more likely to report excellent or very good health compared to heterosexuals and found no significant difference in functional limitations or impairments [13]. However, a limitation is that many of these studies have small sample sizes or combine gay/lesbian with bisexual individuals into one category, and few research has examined the health of middle-aged and older LGBT adults through a multimorbidity framework.

Adults with medical multimorbidity, generally defined as ≥2 concurrent chronic conditions, have high rates of healthcare utilization and often receive poorly coordinated care [14, 15]. Chronic medical diseases, mental illness, and substance use frequently are interrelated and represent a compound multimorbidity placing individuals at even higher risk for adverse outcomes including increases in healthcare utilization [16]. Given that previous research shows high rates for mental illness, drug use, and medical multimorbidity among older LGB adults [3, 9], the goal of this current study was to examine differences regarding these three elements as well as the prevalence of having 2 or 3 concomitant problems using data from a nationally representative sample. As the population of older sexual minorities increases [9, 17], the objective for this study is to better understand the complexity of LGB adults’ health through a multimorbidity lens to help inform multidisciplinary clinical interventions in order to reduce health disparities in this population.

## Methods

### Study population

Aggregated data from the 2015–2017 National Survey on Drug Use and Health (NSDUH) were analyzed. NSDUH is an ongoing cross-sectional survey of non-institutionalized individuals in the 50 US states and the District of Columbia [18]. Data are derived from nationally representative probability samples of populations living in households, noninstitutional group quarters, and shelters, obtained through four sampling stages. Surveys were administered via computer-assisted interviewing conducted by an interviewer and audio computer-assisted self-interviewing. Sample weights were provided by NSDUH to address unit- and individual-level non-response. Weights were adjusted to ensure that estimates were consistent with estimates provided by the US Census Bureau. Further details regarding sampling and survey methods can be found elsewhere [19]. The weighted interview response rates were 69.7% (2015), 68.4% (2016), and 67.1% (2017). This study focuses on adults age ≥ 50 (*n* = 25,880).

### Study measures

#### Sexual orientation and sexual minority status

Participants were asked, “Which one of the following do you consider yourself to be?” and answer options were “heterosexual, that is, straight,” “lesbian or gay,” and “bisexual.” Participants could also report that they do not know or refuse to answer.

#### Drug use

Participants were asked about past-year use of various drugs. In these analyses, we considered past-year self-reported use of cannabis (among those reporting it was never recommended by a doctor), any other illegal drug use (i.e., cocaine, lysergic acid diethylamide [LSD], ecstasy, methamphetamine, ketamine, gamma hydroxybutyrate [GHB], phencyclidine [PCP], N, N-dimethyltryptamine [DMT], heroin), and misuse of prescription pain-relievers (prescription opioids) and tranquilizers (e.g., benzodiazepines aside from flurazepam, temazepam, and triazolam; muscle relaxants). Misuse was defined as using without one’s own prescription; using in greater amounts, more often, or for longer than directed; or use in any way not directed by a doctor. Participants were shown images of various opioid and tranquilizer pills to aid in their recall. We created a variable indicating past-year drug use, which included nonmedical cannabis use, illegal drug use, and prescription drug misuse to encompass the range of potentially unhealthy drug use (which could increase the risk for health consequences).

#### Mental illness

NSDUH includes an indicator for past-year mental illness developed and validated by the Substance Abuse and Mental Health Services Administration and the National Institute of Mental Health, which is based on responses to a list of questions asked by NSDUH. The items include level of emotional distress, functional impairment due to emotional distress, suicidal thoughts, and major depression. Based on the responses an individual is given a dichotomous (yes/no) value for mild, moderate, and severe mental illness. Our definition for mental illness included meeting criteria for moderate or severe mental illness, which is equivalent to Global Assessment of Functioning (GAF) scores of < 60 (serious mental illness is GAF < 50; moderate mental illness is 50 ≤ GAF < 60) [20].

#### Medical multimorbidity

Participants were asked if they had ever had a doctor diagnose them with the following medical conditions: asthma, bronchitis/chronic obstructive pulmonary disease (COPD), cancer, cirrhosis, diabetes, heart conditions, hepatitis B or C, high blood pressure, HIV/AIDS, and kidney disease. Similar to previous studies, we created a variable indicating medical disease multimorbidity which was defined as ≥2 chronic conditions reported [14, 15].

#### Composite of co-occurring conditions

We created a variable indicating the total number of the three interrelated conditions: any past-year drug use, any past-year mental illness, and medical multimorbidity. The composite was coded into 0, 1, and 2–3 conditions. Three conditions were combined with two conditions due to the low prevalence of those reporting all three outcomes.

#### Covariates

We included the following sociodemographic variables as covariates: age group (categorized by NSDUH as 50–64, ≥65), gender, race/ethnicity, education, annual family income, marital status, and whether they receive government assistance.

#### Statistical analysis

Similar to other studies focused on sexual minorities, all analyses were stratified by gender [2, 3]. First, we compared socio-demographic characteristics as well as specific drug use, mental illness, and medical multimorbidity characteristics according to sexual minority status for descriptive purposes. Comparisons were made using Rao-Scott chi-square as this bivariable test takes into account for the complex survey design [21]. We then compared the co-occurring outcome composite variable by heterosexual, lesbian, gay, and bisexual adults in a similar manner. After examining bivariable associations we examined whether sexual minority status was related to reporting drug use, mental illness, and medical multimorbidity in a multivariable manner. Specifically, we examined whether sexual minority status was related to each outcome in three separate binary logistic regression models. Then, using multinomial logistic regression, we determined whether sexual minority status was related to reporting one condition, and 2–3 conditions, compared to reporting no conditions. Since only 413 participants reported “don’t know” or “refuse” as a response for the sexual orientation item, these cases were excluded from the main analyses which focused on the 11,876 men and 14,004 women identifying as heterosexual, gay/lesbian, or bisexual. However, we also conducted a supplemental analysis in which we included those who reported “don’t know” or “refuse” as a response in multivariable models (men *n* = 12,025, female *n* = 14,268). All models controlled for all socio-demographic covariates described above. We used sampling weights (provided by NSDUH) to account for selection probability, non-response, and population distribution, and data were analyzed using survey “svy” commands in Stata SE 13 (StataCorp LP, College Station, TX) which allowed us to account for the complex survey design [21]. We used imputation-revised variables when available to limit missing data as specified and provided by NSDUH [19]. Secondary analysis of these data was exempt for review by our Institutional Review Board.

## Results

Most (97.0%) male participants identified as heterosexual, with 1.9% identifying as gay, and 1.1% identifying as bisexual. Similarly, 97.9% of women identified as heterosexual, 1.2% identified as lesbian, and 0.8% identified as bisexual. As is shown in Table 1, we estimate differences in demographics, drug use, mental illness, and chronic disease between older LGB respondents compared to their heterosexual counterparts stratified by gender. LGB individuals were younger with a higher percentage not married than heterosexuals. Gay men had the highest percentage with an earned a college degree. With regard to past-year drug use, bisexual women had the highest prevalence of nonmedical use of cannabis and misuse of opioids in the past year, with gay men having the highest prevalence of tranquilizers misuse. Bisexual men had the highest prevalence of mental illness and gay men reported the highest prevalence of HIV diagnosis, Hepatitis B or C diagnosis, and having 2 or more chronic diseases.

**Table 1:** Sociodemographic, Drug Use, Mental Illness, and Chronic Disease Characteristics by Sexual Orientation among Adults Age ≥ 50, United States, 2015–2017

	Men				Women			
	Total (*n* = 11,876) % (SE)	Hetero-sexual (*n* = 11,509), % (SE)	Gay (*n* = 227) % (SE)	Bisexual (*n* = 140) % (SE)	Total (*n* = 14,004) % (SE)	Hetero-sexual (*n* = 13,708) % (SE)	Lesbian (*n* = 173) % (SE)	Bisexual (*n* = 123) % (SE)
Survey Year								
2015	33.0 (0.7)	33.1 (0.7)	31.0 (4.3)	32.5 (5.5)	32.8 (0.5)	32.7 (0.5)	33.5 (5.3)	40.4 (5.1)
2016	33.2 (0.5)	33.2 (0.5)	35.1 (3.5)	27.7 (4.4)	33.3 (0.5)	33.4 (0.5)	33.6 (4.8)	20.7 (4.3)
2017	33.8 (0.6)	33.7 (0.6)	33.9 (4.6)	39.8 (5.4)	33.9 (0.4)	33.9 (0.4)	33.0 (4.6)	39.0 (4.6)
Age								
50–64, y	58.4 (0.5) ^b^	58.1 (0.5)	75.0 (3.3)	62.1 (5.6)	54.8 (0.5) ^b^	54.5 (0.6)	66.2 (5.5)	76.3 (4.8)
≥ 65, y	41.6 (0.5)	41.9 (0.6)	25.0 (3.3)	37.9 (5.6)	45.2 (0.5)	45.5 (0.6)	33.8 (5.5)	23.7 (4.8)
Race/Ethnicity								
White	74.2 (0.5)	74.3 (0.5)	69.9 (4.0)	66.3 (4.9)	72.8 (0.6)	72.7 (0.6)	81.4 (3.5)	63.5 (4.6)
Black	9.6 (0.3)	9.5 (0.3)	10.3 (2.0)	11.3 (3.4)	11.1 (0.4)	11.1 (0.4)	7.0 (2.6)	9.7 (3.4)
Hispanic	10.4 (0.4)	10.3 (0.4)	14.8 (3.3)	10.5 (4.0)	10.1 (0.4)	10.1 (0.4)	6.8 (2.2)	16.0 (4.1)
Asian/Other	5.9 (0.3)	5.9 (0.3)	5.1 (2.6)	11.8 (4.1)	6.1 (0.3)	6.0 (0.3)	4.8 (2.0)	10.8 (3.9)
Education								
Less than High School	13.7 (0.4) ^c^	13.8 (0.4)	9.8 (2.1)	11.2 (2.9)	13.8 (0.4) ^c^	13.8 (0.4)	11.3 (2.9)	18.4 (3.5)
High School Diploma	26.4 (0.5)	26.8 (0.5)	11.6 (2.5)	17.4 (4.0)	27.1 (0.5)	27.4 (0.5)	11.2 (2.9)	16.4 (3.8)
Some College	25.8 (0.5)	25.9 (0.6)	25.9 (3.6)	20.8 (4.1)	29.5 (0.5)	29.4 (0.5)	33.0 (4.2)	34.0 (5.1)
College Degree	34.1 (0.6)	33.5 (0.6)	52.7 (4.6)	50.6 (4.1)	29.7 (0.7)	29.5 (0.7)	44.5 (5.0)	31.3 (4.5)
Annual Family Income								
< $20,000	13.2 (0.4) ^a^	13.0 (0.4)	15.8 (2.5)	25.9 (4.9)	17.8 (0.5)	17.8 (0.5)	18.5 (4.2)	18.5 (4.8)
$20,000–$49,999	28.0 (0.5)	28.0 (0.5)	28.3 (4.0)	28.6 (4.7)	31.9 (0.5)	31.9 (0.5)	28.9 (4.0)	36.0 (5.6)
$50,000–$74,999	17.0 (0.4)	17.0 (0.5)	21.5 (3.9)	13.2 (3.6)	16.4 (0.3)	16.4 (0.4)	19.4 (3.2)	16.2 (4.1)
≥ $75,000	41.8 (0.6)	42.0 (0.6)	34.4 (4.8)	32.3 (4.6)	33.9 (0.5)	33.9 (0.5)	33.3 (4.2)	29.4 (4.6)
Relationship Status								
Not Married	30.7 (0.5) ^c^	29.6 (0.4)	75.4 (3.4)	59.0 (5.2)	44.4 (0.6) ^c^	44.0 (0.6)	63.9 (5.6)	64.2 (4.3)
Married	69.3 (0.5)	70.4 (0.4)	24.6 (3.4)	41.0 (5.2)	55.6 (0.6)	56.0 (0.6)	36.1 (5.6)	35.8 (4.3)
Government Assistance	13.7 (0.4)	13.6 (0.4)	18.2 (3.5)	15.5 (3.2)	17.5 (0.4)	17.4 (0.4)	21.2 (4.1)	25.2 (4.3)
Past-Year Drug Use								
Any Past-Year Drug Use	10.5 (0.3) ^c^	10.2 (0.3)	20.9 (4.6)	20.4 (4.7)	6.7 (0.2) ^c^	6.5 (0.2)	13.1 (2.9)	27.7 (4.4)
Cannabis (unprescribed)	7.4 (0.3) ^a^	7.2 (0.3)	13.4 (4.1)	14.4 (3.8)	4.1 (0.2) ^c^	3.9 (0.2)	9.2 (3.0)	21.2 (4.1)
Opioids (misuse)	2.8 (0.2) ^a^	2.7 (0.2)	5.6 (1.9)	5.3 (1.7)	2.1 (0.1) ^b^	2.0 (0.1)	1.8 (1.1)	6.6 (2.2)
Tranquilizers (misuse)	1.2 (0.1) ^c^	1.1 (0.1)	5.4 (2.0)	4.2 (2.1)	1.1 (0.1)	1.1 (0.1)	2.1 (1.4)	1.7 (1.2)
Illegal Drugs (non-cannabis)	1.5 (0.1) ^a^	1.5 (0.1)	2.7 (1.4)	4.8 (2.0)	0.7 (0.1) ^b^	0.7 (0.1)	0.8 (0.8)	4.1 (2.3)
Mental Illness (Past Year)								
Mental illness ^d^	4.8 (0.2) ^c^	4.5 (0.2)	9.8 (2.4)	16.8 (3.4)	7.9 (0.3) ^b^	7.8 (0.3)	8.7 (2.6)	17.8 (4.3)
Chronic Disease								
Asthma	5.8 (0.3)	5.7 (0.3)	8.7 (2.3)	6.3 (2.8)	9.8 (0.3)	9.7 (0.3)	15.0 (3.3)	13.1 (3.7)
Bronchitis/COPD	6.0 (0.3)	6.0 (0.3)	8.2 (1.9)	8.6 (2.5)	9.0 (0.3) ^a^	8.9 (0.3)	15.6 (3.9)	12.0 (3.4)
Cancer	10.7 (0.4)	10.7 (0.4)	12.6 (3.1)	8.1 (2.4)	12.5 (0.3)	12.5 (0.4)	13.2 (3.5)	13.9 (3.9)
Cirrhosis	0.7 (0.1)	0.7 (0.1)	0.8 (0.8)	0.0 (0.0)	0.4 (0.1)	0.4 (0.1)	0.0 (0.0)	2.0 (1.4)
Diabetes	18.4 (0.5)	18.4 (0.5)	19.8 (3.3)	13.5 (3.0)	16.8 (0.4)	16.9 (0.4)	18.2 (3.4)	10.9 (2.6)
Heart Condition	22.1 (0.6)	22.1 (0.5)	22.4 (3.2)	23.5 (4.4)	15.9 (0.4)	15.9 (0.4)	12.4 (2.8)	11.5 (3.2)
Hepatitis B/C	3.0 (0.2) ^c^	2.8 (0.2)	13.6 (2.9)	7.7 (2.6)	1.5 (0.1) ^a^	1.4 (0.1)	2.3 (1.3)	4.9 (2.0)
High Blood Pressure	31.2 (0.5)	31.1 (0.5)	35.7 (3.9)	29.6 (4.8)	36.3 (0.5) ^a^	36.4 (0.5)	29.5 (3.7)	27.2 (4.1)
HIV/AIDS	0.3 (0.1) ^c^	0.7 (0.0)	12.2 (2.6)	1.7 (0.9)	0.1 (0.0)	0.1 (0.0)	0.0 (0.0)	0.0 (0.0)
Kidney Disease	3.3 (0.2)	3.2 (0.2)	4.0 (1.8)	5.3 (2.6)	3.5 (0.2)	3.5 (0.2)	3.1 (1.6)	3.7 (2.0)
0–1 Chronic Diseases	74.3 (0.5) ^b^	74.5 (0.5)	63.3 (3.9)	74.7 (4.1)	73.2 (0.5)	73.2 (0.5)	72.8 (4.0)	74.9 (4.4)
Multiple Chronic Diseases	25.7 (0.5)	25.5 (0.5)	36.7 (3.9)	25.3 (4.1)	26.8 (0.5)	26.8 (0.5)	27.2 (4.0)	25.1 (4.4)

White and black participants were identified as non-Hispanic; *SE* Standard error, *COPD* Chronic obstructive pulmonary disease

^a^Significant at *P* < .05 based on Rao-Scott chi-square test

^b^Significant at *P* < .01 based on Rao-Scott chi-square test

^c^Significant at *P* < .001 based on Rao-Scott chi-square test

^d^Defined as moderate or severe mental illness based on National Survey on Drug Use and Health (NSDUH) indicator [19]

In our adjusted models as shown in Table 2, gay men had a higher odds of having multiple chronic medical conditions (adjusted odds ratio [aOR] = 2.18, 95% confidence interval [CI] = 1.48, 3.21) while no significant difference was found for bisexual men and lesbian/bisexual women compared to their heterosexual counterparts. For mental illness, gay men (aOR = 1.79, 95% CI = 1.09, 2.93) and bisexual men (aOR = 3.53, 95% 2.03, 6.14) had a higher odds of mental illness compared to heterosexual men, while bisexual women had a higher odds of mental illness (aOR = 1.94, 95% CI = 1.03, 3.67) compared to heterosexual women. For drug use, bisexual women had a higher odds of reporting past-year drug use (aOR = 4.20, 95% CI = 2.55, 6.93) compared to heterosexual women, while there were no significant differences between gay and bisexual men and lesbian women and their heterosexual counterparts. With all else being equal, as shown in Table 3, compared to heterosexual men, gay men were nearly three times the odds (aOR = 2.95, 95% CI = 1.60, 5.46) and bisexual men were at more than double the odds (aOR = 2.84, 95% CI = 1.58, 5.08) of reporting 2–3 conditions. Compared to heterosexual women, lesbian women were at more than three times the odds (aOR = 3.25, 95% = 1.60, 6.62) of reporting 2–3 conditions.

**Table 2:** Multivariable Associations between Sexual Orientation and Specific Conditions

**Men**	**Past-Year Drug Use** **aOR (95% CI)**	**≥ 2 Chronic Medical Conditions** **aOR (95% CI)**	**Mental Illness** **aOR (95% CI)**
Heterosexual	Reference group	Reference group	Reference group
Gay	1.62 (0.89, 2.95)	2.18 (1.48, 3.21) ^b^	1.79 (1.09, 2.93) ^a^
Bisexual	1.88 (0.99, 3.56)	1.07 (0.66, 1.72)	3.53 (2.03, 6.14) ^b^
**Women**	**Past-Year Drug Use** **aOR (95% CI)**	**≥ 2 Chronic Medical Conditions** **aOR (95% CI)**	**Mental Illness** **aOR (95% CI)**
Heterosexual	Reference group	Reference group	Reference group
Lesbian	1.70 (0.97, 2.98)	1.05 (0.71, 1.55)	0.87 (0.44, 1.73)
Bisexual	4.20 (2.55, 6.93) ^b^	1.04 (0.64, 1.68)	1.94 (1.03, 3.67) ^a^

Results were derived from six separate multivariable binary logistic regressions controlling for demographic characteristics. The comparison group in each model is no past-year drug use, not having multiple chronic diseases, and not having a mental health disorder in the past year, respectively. *aOR* Adjusted odds ratio, *CI* Confidence intervals, ^a^ Significant at *P* < .05, ^b^ Significant at *P* < .001

**Table 3:** Multivariable Associations between Sexual Orientation and Number of Conditions^a^

**Men**	**1 Condition** **aOR (95% CI)**	**2–3 Conditions** **aOR (95% CI)**
Heterosexual	Reference group	Reference group
Gay	1.74 (1.14, 2.66) ^b^	2.95 (1.60, 5.46) ^c^
Bisexual	1.48 (0.94, 2.31)	2.84 (1.58, 5.08) ^c^
**Women**	**1 Condition** **aOR (95% CI)**	**2–3 Conditions** **aOR (95% CI)**
Heterosexual	Reference group	Reference group
Lesbian	1.03 (0.70, 1.53)	1.38 (0.67, 2.83)
Bisexual	1.63 (1.01, 2.62) ^b^	3.25 (1.60, 6.62) ^c^

Results were derived from multivariable multinomial logistic regressions controlling for demographic characteristics. The comparison group in each model is 0 conditions. *aOR* Adjusted odds ratio, *CI* Confidence intervals, ^a^ Conditions include past-year drug use, multiple chronic medical conditions, and mental illness, ^b^ Significant at *P* < .05, ^c^ Significant at *P* < .01

Finally, in our supplemental analyses we found that all estimates were nearly identical for gay/lesbian and bisexual individuals when including those responding “don’t know” or “refuse” to the sexual orientation question. However, men and women responding “don’t know” were at lower odds for having ≥2 chronic medical conditions, and women who refused to respond were at lower odds for having ≥2 chronic medical conditions and for having a mental illness (Supplemental Table 1). In addition, men responding, “don’t know” and women refusing to respond were at lower odds for having one chronic condition (Supplemental Table 2).

## Discussion

To our knowledge, this is one of few studies of LGB disparities among a nationally representative sample of older adults in the US that examines multimorbidity and co-occurring conditions. We found that for both genders, middle-aged and older LGB adults had higher odds of having 2 or more co-occurring conditions (mental illness, drug use, or medical multimorbidity) compared to heterosexuals. These findings support results from other studies that identify several disparities in health and health-related risk factors among sexual minorities in the US. There are many social determinants that affect the health of sexual minorities in the US that fit with the minority stress model [22], where chronic stressors such as identity concealment, discrimination, and oppression [8], can lead to both mental illness and substance use [23], and ultimately to chronic medical disease. In addition, older LGB adults face the additional challenges of aging that increases the risks for chronic medical disease and social isolation [24]. Older LGB adults in particular often have barriers to social services and culturally competent healthcare providers [25]. Therefore older LGB adults are at elevated health risks and interventions focused on reducing the risk of poor health outcomes and a premature decline in function in this population should consider these conditions that may occur simultaneously.

An abundance of literature suggests that rates of substance use and SUDs are higher among LGBT persons compared to heterosexuals [11, 26, 27]; however, there remains very little data on prevalence among middle-aged and older adults. Our study is one of few that reports drug use patterns among older LGB adults especially by gender and sexual orientation. A recent study of aggregated LGB adults age 50 and older found a higher odds of nonmedical cannabis use and prescription misuse of opioids and tranquilizers [12]. For this study, we found a much higher prevalence of nonmedical use of cannabis specifically among bisexual women. In addition, bisexual women had the highest prevalence of prescription opioid misuse compared to all other groups. Gay men meanwhile had the highest prevalence of prescription tranquilizer misuse, and bisexual men had the highest prevalence of illegal drug use. More research is needed to understand patterns of substance use among middle-aged and older LGB adults as factors for increased drug use likely differ from younger LGB adults, who may be more likely to use “club drugs” (i.e. methamphetamine, ecstasy) than older LGB individuals [28, 29]. Several reasons may explain drug use in this population including stressors due to minority status, social isolation, as well as mental illness.

In this study, both male and female sexual minorities had a higher prevalence of mental illness compared to heterosexuals, with bisexual women having the highest prevalence. This also may be due to exposure to chronic stressors over the life course including identity concealment and the dual stigmatization of aging and belonging to a sexual minority group (and for some, also belonging to a racial/ethnic minority group). Our findings are largely consistent with several studies both in the US and in Canada that demonstrate the higher prevalence of depression, suicide attempts, and poorer mental health among older LGB compared to heterosexuals [9, 30–32]. The highest prevalence among bisexual men and women in our study may be due to lower levels of community connectedness compared to lesbian and gay men [9], which may increase isolation as one ages.

In terms of chronic disease and medical multimorbidity, our study adds new information regarding the health of middle-age and older adults among LGB adults. However, previous work on chronic disease burden in this population using national data combines adults identifying as bisexual and as gay or lesbian [3], and therefore it is difficult to make comparisons. Our study results found that gay men had the highest prevalence of multiple chronic diseases, possibly driven, in part, by also having the highest prevalence of HIV/AIDS. While older bisexual women had a lower prevalence of medical multimorbidity, they were at increased risk for having more than one co-occurring condition. Therefore, although bisexual women may appear to have a more “favorable” medical multimorbidity profile if examined in isolation, they are in fact still at high risk for poor health outcomes through multiple co-existing factors. This is consistent with previous research in younger populations, which has found that bisexual women were less likely to engage in health-related behaviors, report poor health status or disability, use smokeless tobacco and opioids, and not seek healthcare because of cost [1, 33, 34]. Further work is needed to better understand the health needs of older bisexual women to reduce sexual minority health disparities.

The compound multimorbidity of having more than one condition (drug use, mental illness, and/or medical multimorbidity) has implications for better understanding determinants of health for older sexual minorities and how to develop clinical interventions to address care in the setting of complexity. One method of studying such multimorbidity is a syndemic framework with a focus on how conditions are interconnected and adversely affected by behavioral and psychosocial conditions [35–37]. Syndemic theory describes how two or more conditions interact synergistically to contribute to increased vulnerability and disease burden [38], and has been used commonly to describe risks among sexual minority groups [37, 39], Our results inform the use of syndemic theory for future research in this population as older sexual minorities are at higher risk for having co-occurring conditions, which are often interrelated. Future studies could use the syndemic framework to better delineate profiles of risk by examining synergistic interactions between conditions. In addition, for all older sexual minorities, future research needs to focus on how specific conditions and co-occurring conditions predict poor health outcomes and functional decline. Lastly, future research can adopt an intersectional lens [40] to further examine subgroup differences in sexual minority disparities for multimorbidity, including racial/ethnic and nativity differences regarding co-occurring conditions among older adults by gender, which is a priority [8].

### Limitations

Self-report is a limitation, as such data are susceptible to limited recall and social desirability bias. In addition, NSDUH does not ask whether participants are transgender and studies are increasingly showing health disparities among gender minorities. Another limitation of our study is that 413 participants responded “don’t know” or “refuse” for the sexual orientation item. Our supplemental analyses including those responding to these categories suggest there is the possibility of bias for the outcomes of chronic medical conditions and mental illness. It is unknown which of these respondents legitimately do not know their identity, and it is unknown who actually might have identified as “other” sexuality, while others might have merely satisficed and chose such a response without thinking much about it. We highly recommend that surveys asking about sexual orientation provide an option for “other” sexuality [41]. However, the number of respondents not reporting a listed sexual orientation was low (< 1.6%) and does not appear to affect the main outcome of co-occurring conditions. Further, the sample sizes were too small to examine differences between racial/ethnic subgroups of LGB. The NSDUH also samples only the non-institutionalized US population, and therefore does not include adults who live in long-term care settings. Finally, the survey is cross-sectional, and different participants were sampled each year; therefore, this study cannot establish causality.

## Conclusions

Older sexual minorities in the US are at heightened risk for the interrelated and compound morbidity from mental illness, drug use, and chronic medical diseases. These disparities are likely due to minority stressors and discrimination along with aging and increasing social isolation. These findings indicate the need to consider how co-occurring conditions can contribute to poor health outcomes in older sexual minority populations and the need for specific interventions within this diverse group.

## Supplementary information

**Additional file 1: Supplemental Table 1** Sensitivity Tests Examining Multivariable Associations between Sexual Orientation and Specific Conditions. Results were derived from six separate multivariable binary logistic regressions controlling for demographic characteristics and include categories indicating responses to the sexual orientation question of “don’t know” and “refused.” The comparison group in each model is no past-year drug use, not having multiple chronic diseases, and not having a mental health disorder in the past year, respectively. aOR = Adjusted odds ratio, CI = confidence intervals, ^a^ Significant at *P* < .05, ^b^ Significant at *P* < .001.

**Additional file 2: Supplemental Table 2** Sensitivity Tests Examining Multivariable Associations between Sexual Orientation and Number of Conditions^a.^ Results were derived from multivariable multinomial logistic regressions controlling for demographic characteristics and include categories indicating responses to the sexual orientation question of “don’t know” and “refused.” The comparison group in each model is 0 conditions. aOR = Adjusted odds ratio, CI = confidence intervals, ^a^ Conditions include past-year drug use, multiple chronic medical conditions, and mental illness, ^b^ Significant at *P* < .05, ^c^ Significant at *P* < .01.

## Data Availability

The datasets used from the National Survey on Drug Use and Health for this study are publicly available at: https://www.datafiles.samhsa.gov/study-series/national-survey-drug-use-and-health-nsduh-nid13517

## Abbreviations

aOR: Adjusted odds ratio
CI: Confidence interval
GAF: Global Assessment of Functioning
LGB: Lesbian, gay, and bisexual
LGBT: Lesbian, gay, bisexual, and transgender
NHIS: National Health Interview Survey
NSDUH: National Survey on Drug Use and Health
SUD: Substance use disorder
US: United States
