# Supplementary material for: Co-occurring mental illness, drug use, and medical multimorbidity among lesbian, gay, and bisexual middle-aged and older adults in the United States: a nationally representative study
Source: BMC Public Health. 2020 Aug 4;20:1123. doi: 10.1186/s12889-020-09210-6 (PMC7401198; doi:10.1186/s12889-020-09210-6)
Supplement: Supplementary file 1 — Additional file 1: Supplemental Table 1 Sensitivity Tests Examining Multivariable Associations between Sexual Orientation and Specific Conditions. Results were derived from six separate multivariable binary logistic regressions controlling for demographic characteristics and include categories indicating responses to the sexual orientation question of “don’t know” and “refused.” The comparison group in each model is no past-year drug use, not having multiple chronic diseases, and not having a mental health disorder in the past year, respectively. aOR = Adjusted odds ratio, CI = confidence intervals, a Significant at P < .05, b Significant at P < .001. [file 12889_2020_9210_MOESM1_ESM.docx]

SUPPLEMENTAL TABLE 1—Sensitivity Tests Examining Multivariable Associations between Sexual Orientation and Specific Conditions

| Men | Past-Year Drug Use  aOR (95% CI) | >2 Chronic Medical Conditions  aOR (95% CI) | Mental Illness  aOR (95% CI) |
| --- | --- | --- | --- |
| Heterosexual | Reference group | Reference group | Reference group |
| Gay | 1.62 (0.89, 2.95) | 2.17 (1.47, 3.20) ^b^ | 1.79 (1.09, 2.93) ^a^ |
| Bisexual | 1.88 (0.99, 3.56) | 1.07 (0.66, 1.72) | 3.54 (2.03, 6.17) ^b^ |
| Don’t Know | 1.04 (0.29, 3.69) | 0.07 (0.01, 0.56) ^a^ | 1.37 (0.33, 5.66) |
| Refused | 0.60 (0.25, 1.48) | 0.72 (0.36, 1.43) | 0.60 (0.18, 2.01) |
| Women | Past-Year Drug Use  aOR (95% CI) | >2 Chronic Medical Conditions  aOR (95% CI) | Mental Illness  aOR (95% CI) |
| Heterosexual | Reference group | Reference group | Reference group |
| Lesbian | 1.69 (0.96, 2.96) | 1.06 (0.72, 1.56) | 0.87 (0.44, 1.72) |
| Bisexual | 4.23 (2.57, 6.96) ^b^ | 1.05 (0.65, 1.70) | 1.95 (1.04, 3.67) ^a^ |
| Don’t Know | 1.09 (0.32, 3.73) | 0.30 (0.12, 0.71) ^b^ | 0.85 (0.22, 3.26) |
| Refused | 0.64 (0.21, 1.90) | 0.46 (0.25, 0.86) ^a^ | 0.29 (0.09, 0.89) ^a^ |

Results were derived from six separate multivariable binary logistic regressions controlling for demographic characteristics and include categories indicating responses to the sexual orientation question of “don’t know” and “refused.” The comparison group in each model is no past-year drug use, not having multiple chronic diseases, and not having a mental health disorder in the past year, respectively. aOR=Adjusted odds ratio, CI=confidence intervals, ^a^ Significant at *P* < .05, ^b^ Significant at *P* < .001
