# Supplementary material for: Co-occurring mental illness, drug use, and medical multimorbidity among lesbian, gay, and bisexual middle-aged and older adults in the United States: a nationally representative study
Source: BMC Public Health. 2020 Aug 4;20:1123. doi: 10.1186/s12889-020-09210-6 (PMC7401198; doi:10.1186/s12889-020-09210-6)
Supplement: Supplementary file 2 — Additional file 2: Supplemental Table 2 Sensitivity Tests Examining Multivariable Associations between Sexual Orientation and Number of Conditionsa. Results were derived from multivariable multinomial logistic regressions controlling for demographic characteristics and include categories indicating responses to the sexual orientation question of “don’t know” and “refused.” The comparison group in each model is 0 conditions. aOR = Adjusted odds ratio, CI = confidence intervals, a Conditions include past-year drug use, multiple chronic medical conditions, and mental illness, b Significant at P < .05, c Significant at P < .01. [file 12889_2020_9210_MOESM2_ESM.docx]

SUPPLEMENTAL TABLE 2—Sensitivity Tests Examining Multivariable Associations between Sexual Orientation and Number of Conditions^a^

| Men | 1 Condition  aOR (95% CI) | 2-3 Conditions  aOR (95% CI) |
| --- | --- | --- |
| Heterosexual | Reference group | Reference group |
| Gay | 1.74 (1.14, 2.65) ^b^ | 2.96 (1.60, 5.48) ^b^ |
| Bisexual | 1.47 (0.94, 2.31) | 2.84 (1.59, 5.09) ^b^ |
| Don’t Know | 0.30 (0.10, 0.88) ^a^ | 0.69 (0.09, 5.35) |
| Refused | 0.66 (0.36, 1.22) | 0.16 (0.02, 1.22) |
| Women | 1 Condition  aOR (95% CI) | 2-3 Conditions  aOR (95% CI) |
| Heterosexual | Reference group | Reference group |
| Lesbian | 1.03 (0.70, 1.53) | 1.38 (0.68, 2.83) |
| Bisexual | 1.64 (1.02, 2.65) ^a^ | 3.28 (1.61, 6.68) ^c^ |
| Don’t Know | 0.45 (0.19, 1.08) | 0.45 (0.06, 3.25) |
| Refused | 0.36 (0.23, 0.58) ^c^ | 0.40 (0.09, 1.83) |

Results were derived from multivariable multinomial logistic regressions controlling for demographic characteristics and include categories indicating responses to the sexual orientation question of “don’t know” and “refused.” The comparison group in each model is 0 conditions. aOR=Adjusted odds ratio, CI=confidence intervals, ^a^ Conditions include past-year drug use, multiple chronic medical conditions, and mental illness, ^b^ Significant at *P* < .05, ^c^ Significant at *P* < .01
